# Equilibrium Studies of Dibutyltin(IV)–Zwitterionic Buffer Complexation

**DOI:** 10.1007/s10953-013-0088-5

**Published:** 2013-10-26

**Authors:** M. A. El-Gahami, H. M. Albishri

**Affiliations:** 1 Department of Chemistry, Faculty of Science, King Abdulaziz University, P.O. Box 8020, North Jeddah, Kingdom of Saudi Arabia; 2 Department of Chemistry, Faculty of Science, Assiut University, Assiut, Egypt

**Keywords:** Equilibrium studies, Dibutyltin(IV) complexes, “Good’s buffers”, Effect of solvent and temperature, Thermodynamic parameters

## Abstract

Equilibrium studies in aqueous solution are reported for dibutyltin(IV) (DBT) complexes of the zwitterionic buffers “Good’s buffers” Mes and Mops. Stoichiometric and formation constants of the complexes formed were determined at different temperatures and ionic strength 0.1 mol·L^−1^ NaNO_3_. The results show that the best fit of the titration curves were obtained when the complexes ML, MLH_−1_, MLH_−2_ and MLH_−3_ were considered beside the hydrolysis product of the dibutyltin(IV) cation. The thermodynamic parameters Δ*H* ^o^, Δ*S* ^o^ and Δ*G* ^o^ calculated from the temperature dependence of the formation constant of the dibutyltin(IV) complexes with 2-(*N*-morpholino)ethanesulfonic acid (Mes) and 3-(*N*-mor-pholino)-propanesulfonic acid (Mops) were investigated. The effect of dioxane as a solvent on the formation constants of DBT–Mes and DBT–Mops complexes decrease linearly with the increase of dioxane proportion in the medium. The concentration distribution of the various complexes species was evaluated as a function of pH.

## Introduction

Organotin(IV) compounds are receiving increasing interest because of their biological and potential pharmaceutical applications as antitumor agents [1–6]. On the other hand, in recent years many organotin(IV) compounds have been tested for their in vitro activity against a large variety of tumor lines and have been found to be as effective as, or better than, traditional heavy metal anticancer drugs such as *cis*-platin [6]. In addition, organotin(IV) compounds are also of interest in view of their considerable structural diversity.

Zwitterionic buffers [7–9] are currently used routinely in biochemical and speciation studies under the assumption that they undergo little if any interaction with biologically (or environmentally) important metal ions. However, there has been an increase in reports on buffer interferences when most of Good’s buffers were used in the presence of metal ions [10–16]. Many conflicting data and conclusions that were reported by investigators studying identical metal cation protein systems (at the same pH in carefully executed experiments) might be due in part to the failure to include the presence of metal–buffer complexes.

These compounds (Good’s buffers) contain hydroxyl, carboxylate or sulfydryl groups in addition to a primary nitrogen donor atom. Therefore, one might expect that metal complexes of these buffers would form in biochemical/environmental systems containing metal ions, when these compounds are used to control the pH. On the other hand, it is known that 2-(*N*-morpholino)ethanesulfonic acid (Mes) and 3-(*N*-morpholino)-propanesulfonic acid (Mops) are important hydrogen ion buffers for biological media.

This paper describes an investigation of the different complex species of the two biologically important compounds (dibutyltin(IV), M and zwitterionic buffers, L). A systematic study of this complex system has been carried out. The effect of solvent and temperature on the complex formation equilibrium were also investigated. Stoichiometric and stability of the various complex species in the complex system have been examined by pH-metric techniques.

The structure of the compounds under investigations can be represented diagrammatically as follows:

## Experimental

### Material and Reagents

Dibutyltin(IV) dichloride was received from Aldrich Chemicals Co., Germany (purity 99 %). The ligands (L) used were 2-(*N*-morpholino) ethanesulfonic acid (Mes) and 3-(*N*-morpholino) propanesulfonic acid (Mops); their purities were 98 and 99 %, respectively. These materials were supplied from Flucka Chemicals Co., Germany. Carbonate-free sodium hydroxide stock solutions were prepared by diluting the contents of concentrated volumetric solution vials. These solutions were systematically checked by titration against potassium hydrogen phthalate. These materials (sodium hydroxide, potassium hydrogen phthalate and NaNO_3_) were received from BDH and purified according to literature methods [14, 16]. All solutions were prepared in deionized water.

### Apparatus

pH titrations were performed using a Metrohm 751 GPD titrino instrument. The titroprocessor was calibrated with standard buffer solutions prepared according to [17]. The titrations were carried out in a purified nitrogen atmosphere using a titration vessel described previously [18]. The temperature was maintained constant by a Color Ultra Thermostat.

### Procedure and Measuring Techniques

The protonation constants of the ligands were determined by titrating a 40 mL of ligand solution (2.50 mol·L^−1^). The hydrolysis constants of dibutyltin(IV) were determined by titrating a 40 mL of dibutyltin(IV) dichloride (2.50 mol·L^−1^). The formation constant of organotin(IV) complexes was determined by titrating a 40 mL of solution containing the ligand (2.50 mol·L^−1^) and dibutyltin(IV) with concentrations 2.50 and 1.25 mol·L^−1^. The titrations were performed at different temperatures and in dioxane–water mixtures of different compositions. The ionic strength was adjusted to 0.10 mol·L^−1^ with NaNO_3_. p*K*_w_s at different temperatures and in dioxane–water solutions were determined as described previously [19]. For this purpose, various amounts of standard NaOH solution were added to a solution containing 0.10 mol·L^−1^ NaNO_3_. The [OH^−^] was calculated from the amount of base added. The [H^+^] was calculated from the pH value. The product of [OR] and [H^+^] was taken and the mean values obtained in this way for the log_10_ concentration product are 1og_10_ *K*_w_ = −14.28, −14.72, −15.13, −15.46, −15.70, −16.21 for 12.50, 25.00, 37.50, 50.00, 62.50 and 75.00 % by mass dioxane–water solutions, and log_10_ *K*_w_ = −14.25, −14.07, −13.89, −13.74 and −13.69 for 15.00, 20.00, 25.00, 30.00 and 35.00 °C for water solutions, respectively.

The species formed in the systems were characterized by the general equilibrium processes are given by Eq. 1, while the formation constants for these generalized species are given by Eq. 2:

1 $$ p{\text{M}} + q{\text{L}} + r{\text{H}} \rightleftharpoons \left[ {{\text{M}}_{p} {\text{L}}_{q} {\text{H}}_{r} } \right] $$

2 $$ \beta_{pqr} = \frac{{\left[ {{\text{M}}_{p} {\text{L}}_{q} {\text{H}}_{r} } \right]}}{{\left[ {\left[ {\text{M}} \right]^{p} \left[ {\text{L}} \right]^{q} \left[ {\text{H}} \right]^{r} } \right]}} $$

The calculations were performed using the computer program [20] MINIQUAD-75 loaded on a Pentium II–233 computer. The stoichiometric and stability constants of the complexes formed were determined by trying various possible composition models for the system. The model was selected to give the best statistical fit and be chemically consistent with the titration data without giving any systematic drifts in the magnitudes of various residuals, as described elsewhere [20]. The concentration distribution diagrams were obtained using the program SPECIES [21].

## Results and Discussion

### Solution-Phase Investigations

The dibutyltin(IV) cation is known [19, 22–26] to form stable water soluble mono- and polynuclear hydroxide species (Tables 1 and 2) in the whole pH range studied. Since the hydroxide ion and the ligands are in strong competition for the metal ion, these species were always taken into consideration in the equilibrium systems.

**Table 1:** Formation constants of dibutyltin(IV)–Mes complexes in water at different temperatures

System^a^	Temperature (°C)	*p* ^b^	*q* ^b^	*r* ^b^	log_10_ *β* ^c^	*S* ^d^
DBT	15.00	1	0	−1	−2.50 (0.01)	4.4 × 10^−8^
		1	0	−2	−8.00 (0.01)	
		1	0	−3	−29.50 (0.03)	
		2	0	−4	−9.10 (0.01)	
		2	0	3	−14.21 (0.02)	
DBT–Mes		0	1	1	4.30 (0.01)	2.1 × 10^−8^
		1	1	0	6.51 (0.01)	1.6 × 10^−8^
		1	1	−1	2.16 (0.01)	
		1	1	−2	−3.10 (0.02)	
		1	1	−3	−11.60 (0.01)	
DBT	20.00	1	1	−1	−3.81 (0.01)	4.2 × 10^−8^
		1	1	−2	−7.81 (0.01)	
		1	1	−3	−28.10 (0.01)	
		2	1	−4	−8.91 (0.03)	
		2	1	−2	−13.98 (0.01)	
DBT–Mes		1	1	−1	4.60 (0.01)	1.6 × 10^−8^
		1	1	−2	6.83 (0.01)	1.4 × 10^−8^
		1	1	−3	2.50 (0.01)	
		1	1	−4	−3.61 (0.03)	
		1	1	−2	−12.00 (0.02)	
DBT	25.00	1	0	−1	−4.01 (0.01)	3.7 × 10^−8^
		1	0	−2	−8.30 (0.01)	
		1	0	−3	−28.62 (0.02)	
		1	0	−4	−9.30 (0.01)	
		1	0	−3	−14.61 (0.02)	
DBT–Mes		0	1	−1	4.81 (0.01)	1.3 × 10^−8^
		1	1	−2	7.30 (0.01)	1.0 × 10^−8^
		1	1	−3	3.22 (0.01)	
		1	1	−4	−4.21 (0.02)	
		2	1	−3	−12.45 (0.02)	
DBT	30.00	1	0	5	−4.83 (0.01)	3.3 × 10^−8^
		1	0	−1	−9.21 (0.01)	
		1	0	−2	−28.80 (0.02)	
		2	0	−3	−9.62 (0.03)	
		2	0	−4	−15.00 (0.01)	
DBT–Mes		1	1	−1	5.20 (0.01)	7.3 × 10^−9^
		1	1	−2	7.60 (0.01)	8.8 × 10^−9^
		1	1	−3	3.82 (0.02)	
		1	1	−4	−4.50 (0.03)	
		1	1	−3	−12.88 (0.02)	
DBT	35.00	1	1	−1	−6.20 (0.01)	3.7 × 10^−8^
		1	0	3	−8.30 (0.01)	
		1	0	−1	−25.30 (0.01)	
		2	0	−3	−8.65 (0.02)	
		1	0	−1	14.30 (0.01)	
DBT–Mes		1	1	−4	6.20 (0.01)	
		1	1	−1	8.00 (0.01)	7.9 × 10^−9^
		1	1	−2	−5.20 (0.01)	9.2 × 10^−9^
		1	1	−3	−12.40 (0.01)	
		1	1	−4	−6.00 (0.00)	

^a^Mes is 2-(*N*-morpholino)ethanesulfonic acid

^b^ *p*, *q*, *r* are the stoichiometric coefficient corresponding to dibutyltin(IV), Good’s buffers and H^+^, respectively

^c^Standard deviations are given in parentheses

^d^Sum of square of residuals

**Table 2:** Formation constants of dibutyltin(IV)–Mops complexes in water at different temperature

System	Temperature (°C)	*p*	*q*	*r*	log_10_ *β*	*S*
DBT–Mops	15.00	1	0	1	−3.46 (0.01)	1.8 × 10^−7^
		1	0	2	−8.01 (0.01)	1.3 × 10^−7^
		1	0	−3	−25.40 (0.03)	
		2	0	−2	−4.52 (0.01)	
		2	0	−4	−14.11 (0.01)	
DBT–Mops	20.00	0	0	−1	−3.30 (0.01)	2.4 × 10^−7^
		1	0	−2	−7.60 (0.01)	1.8 × 10^−8^
		1	0	−3	−23.65 (0.02)	
		1	0	−2	−3.38 (0.01)	
		1	0	−4	−12.98 (0.01)	
DBT–Mops	25.00	0	1	1	−3.03 (0.01)	7.5 × 10^−8^
		1	1	0	−6.68 (0.01)	1.5 × 10^−9^
		1	1	−1	−23.06 (0.01)	
		1	1	−2	−3.08 (0.01)	
		1	1	−3	−12.61 (0.01)	
DBT–Mops	30.00	0	1	1	−2.56 (0.01)	8.3 × 10^−8^
		1	1	0	−6.12 (0.03)	2.6 × 10^−9^
		1	1	−1	−22.66 (0.01)	
		1	1	−2	−2.76 (0.01)	
		1	1	−3	−11.51 (0.01)	

The acid dissociation constants of Mes and Mops were determined under the same experimental conditions of ionic strength and temperature (Tables 1 and 2). These constants should be taken into consideration during the evaluation of the pH-metric data. It is found that log_10_ *β*_011_ = 6.06 and 7.12 for Mes and Mops, respectively [27]. The protonation constant for the zwitterionic buffers (Good’s buffers) clearly indicate the different basicity of tertiary amine. This is due to the substitution of increasingly larger alkyl chains between the tertiary amine group and the sulfonate group, the addition of hydroxyl groups on the molecule, or other modifications to the backbone N-substituted structure.

**Fig. 1:**
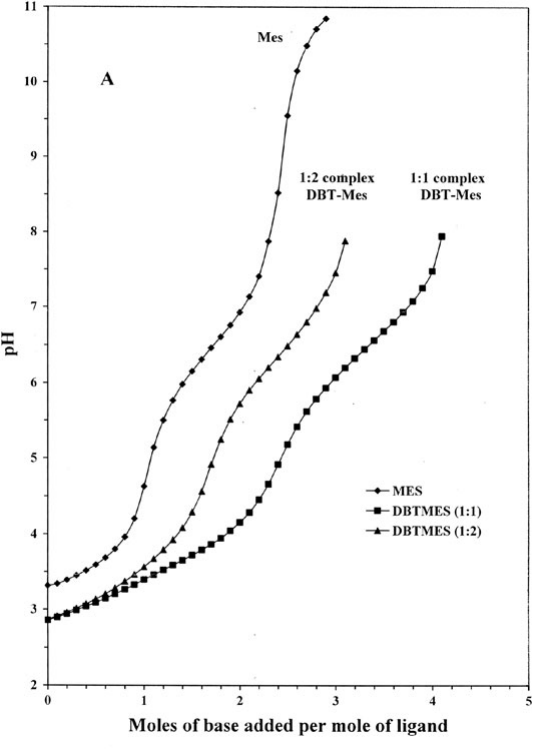
Potentiometric titration curves of DBT–Mes in water

The titration curves for the complex solutions with (1:1 and 2:1) ligand-to-metal ratio clearly indicate the formation of different kinds of complexes (Fig. 1). The best fit of the titration curves was obtained when complexes ML, MLH_−l_, MLH_−2_ and MLH_−3_ were included beside the hydrolysis products of the dibutyltin(IV) cation (Tables 1 and 2).

The potentiometric titration curves of dibutyltin(IV) complexes against both of Mes and Mops with different stoichiometric species (1:1 and 1:2, metal:ligand) of these systems started at pH ~ 3.0. At this pH the sulphonate group was already deprotonated and does not play a role in the mode of coordination with the DBT(IV) anion. With increasing pH Mes and Mops react with DBT(IV) as monodentate ligands forming deprotonated complexes ML. ML competes with the hydroxo complex at lower pH ~ 3.0. At higher pHs further deprotonation was observed leading to form mixed-hydroxo complex species MLH_−1_, MLH_−2_, and MLH_−3_.

### Effect of Temperature

The thermodynamic parameters ΔH*, ΔS* and ΔG* were obtained by a linear least-squares fit of log_10_ *K* versus l/*T* leading to an intercept ΔS*/*R* and a slope −ΔH*/*R*. The results obtained are summarized in Table 3 and explained as follows:

**Table 3:** Thermodynamic parameters for the equilibrium of dibutyltin(IV) complexes in water

Equilibrium	Δ*H* ^o^ (kJ·mol^−1^)	Δ*S* ^o^ (J·K^−1^·mol^−1^)	Δ*G* ^o^ (kJ·mol^−1^)
DBT–Mes			
M^2+^ + L^−^ ⇌ ML^+^	−37.5 (0.30)	−25 (1)	−30.6 (0.6)
ML^+^ + OH^−^ ⇌ MLH_−1_	−58.2 (0.01)	−160 (1)	−20.5 (0.5)
MLH_−1_ + OH^−^ ⇌ MLH_−2_	−25.3 (0.01)	36 (1)	−32.8 (0.3)
MLH_−2_ + OH^−^ ⇌ MLH_−3_	−34.2 (0.03)	230 (1)	−40.1 (0.1)
DBT–Mops			
M^2+^ + L^−^ ⇌ ML^+^	−40.2 (0.30)	−31 (2)	−40.1 (0.3)
ML^+^ + OH^−^ ⇌ MLH_−1_	−65.3 (0.10)	−172 (1)	−25.3 (0.1)
MLH_−1_ + OH^−^ ⇌ MLH_−2_	−36.5 (0.30)	25 (2)	−35.6 (0.5)
MLH_−2_ ⇌ MLH_−3_	−35.2 (0.10)	220 (1)	−50.3 (0.1)

**Fig. 2:**
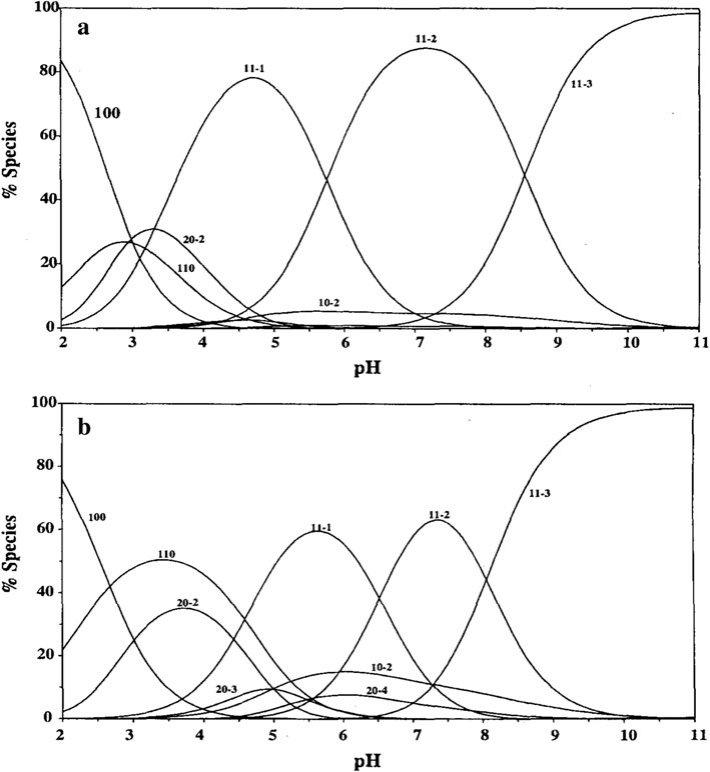
Species distribution curves in the DBT(IV)–Mes (**a**) and DBT(IV)–Mops (**b**) systems. [M] = 2.50 mol·L^−1^, [L] = 2.50 mol·L^−1^. The notation of the different species corresponds to the *pqr* values of the corresponding complex M*p*L*q*H*r*

- The formation constants for the hydrolyzed species of dibutyltin(IV) are discussed in the previous work [19].
- The protonation reactions of the tertiary amine site of Mes are exothermic and the entropy is positive. This means that the protonation constant of Mes is ordered and favored with increasing temperature.
- The complex-formation of Mes with DBT is exothermic for ML, MLH_−1_ and MLH_−2_ species and endothermic for MLH_−3_ species. This may be explained statistically based on the presumption that more coordination sites are available for binding ML, MLH_−1_ and MLH_−2_ species (Fig. 2). The formation of the MLH_−1_ species is more exothermic than for the ML species. This means than the hydroxide complex is more favored than the deprotonated complex species, due to the high affinity of dibutyltin(IV) to form hydroxide complex.

### Effect of Solvent

Traditionally, water has been considered as the solvent which best represents biological conditions. Although this is a general assumption, a lower polarity has been detected in some biochemical micro-environments, such as active sites of enzymes and side chains in proteins, sometimes hidden in lower dielectric constant cavities [28–32]. In these cases, the selection of other solvents seems more appropriate in order to emulate properly the medium’s real features. Careful examination of the medium effect on the equilibrium constants, Table 4, is summarized in the following:

**Table 4:** Formation constants of dibutyltin(IV) complexes in 75.00 mass-% dioxane–25.00 mass-% water solutions at different compositions

System	Dioxane (%)	*p*	*q*	*r*	log_10_ *β*	*S*
DBT	12.50	1	0	−1	−3.1 (0.01)	1.2 × 10^−7^
		1	0	−2	−8.30 (0.01)	
		1	0	−4	−29.01 (0.02)	
		2	0	−2	−3.50 (0.01)	
		2	0	−3	−7.88 (0.02)	
DBT–Mes		0	1	1	5.12 (0.01)	3.1 × 10^−8^
		1	1	0	4.31 (0.01)	2.8 × 10^−9^
		1	1	−1	1.98 (0.01)	
		1	1	−2	−3.40 (0.01)	
		1	1	−3	−10.98 (0.02)	
DBT–Mops		0	1	−1	7.63 (0.01)	3.6 × 10^−8^
		1	1	−2	4.68 (0.01)	2.1 × 10^−9^
		1	1	−3	2.10 (0.01)	
		1	1	−4	−4.61 (0.02)	
		1	1	−2	−12.63 (0.03)	
DBT	25.00	1	0	−1	−3.65 (0.01)	1.0 × 10^−8^
		1	0	−2	−8.87 (0.01)	
		1	0	−3	−30.20 (0.02)	
		2	0	−4	−3.81 (0.03)	
		2	0	−2	−8.20 (0.02)	
DBT–Mes		0	1	1	6.12 (0.01)	4.2 × 10^−8^
		1	1	0	5.30 (0.01)	3.6 × 10^−9^
		1	1	−1	2.12 (0.01)	
		1	1	−2	−4.5 (0.01)	
		1	1	−3	−11.12 (0.03)	
DBT–Mops		1	1	0	7.66 (0.01)	5.68 × 10^−8^
		1	1	−1	6.12 (0.01)	3.8 × 10^−9^
		1	1	−2	3.12 (0.01)	
		1	1	−3	−6.13 (0.02)	
		1	1	−4	−12.60 (0.03)	
DBT	37.50	1	0	−1	−4.20 (0.01)	1.5 × 10^−8^
		1	0	−2	−9.10 (0.01)	
		1	0	−3	−31.0 (0.02)	
		1	0	−4	−4.21 (0.03)	
		2	0	−2	−8.41 (0.03)	
DBT–Mes		1	1	1	6.50 (0.01)	4.1 × 10^−8^
		1	1	0	6.12 (0.01)	2.1 × 10^−9^
		1	1	−1	3.0 (0.02)	
		1	1	−2	4.80 (0.01)	
		1	1	−3	11.50 (0.03)	
DBT − Mops		1	1	0	7.80 (0.01)	4.8 × 10^−8^
		1	1	−1	6.40 (0.01)	3.6 × 10^−9^
		1	1	−2	3.16 (0.02)	
		1	1	−3	−6.90 (0.03)	
		1	1	−4	−13.70 (0.01)	
DBT	50.00	0	1	1	−4.6 (0.01)	1.8 × 10^−8^
		1	1	0	−9.36 (0.01)	
		1	1	−1	−32.10 (0.01)	
		1	1	−2	−4.61 (0.02)	
		1	1	−3	−8.80 (0.01)	
DBT–Mes		1	1	−1	6.80 (0.01)	5.1 × 10^−8^
		1	1	−2	6.88 (0.01)	2.4 × 10^−9^
		2	1	−3	3.61 (0.02)	
		1	1	−4	−5.20 (0.01)	
		1	1	−2	−12.10 (0.03)	
DBT–Mops		1	1	−1	6.85 (0.01)	5.3 × 10^−8^
		1	1	−2	7.10 (0.01)	3.8 × 10^−9^
		1	1	−3	3.91 (0.02)	
		2	1	−4	−5.50 (0.01)	
		2	1	−1	−12.60 (0.03)	
DBT	62.50	1	0	−1	−4.88 (0.01)	2.2 × 10^−8^
		1	0	−2	−9.62 (0.01)	
		1	0	−3	−32.33 (0.02)	
		2	0	−4	−4.31 (0.01)	
		2	0	−2	−9.12 (0.03)	
DBT–Mes		0	1	1	6.88 (0.01)	5.4 × 10^−8^
		1	1	0	7.12 (0.01)	2.8 × 10^−9^
		1	1	−1	3.82 (0.02)	
		1	1	−2	−5.51 (0.01)	
		1	1	−3	−12.31 (0.03)	
DBT–Mops		1	1	1	7.21 (0.01)	6.1 × 10^−8^
		1	1	0	7.56 (0.01)	4.0 × 10^−9^
		1	1	−1	4.20 (0.02)	
		2	1	−2	−5.81 (0.03)	
		2	1	−3	−2.81 (0.02)	
DBT	75.00	1	0	−1	−5.10 (0.01)	2.6 × 10^−8^
		1	0	−2	−9.82 (0.01)	
		1	0	−3	−32.50 (0.02)	
		2	0	−4	−4.66 (0.01)	
		2	0	−2	−9.54 (0.03)	
DBT–Mes		0	1	−1	7.20 (0.01)	6.1 × 10^−8^
		1	1	−2	7.51 (0.01)	3.2 × 10^−9^
		1	1	−3	4.12 (0.01)	
		1	1	−4	−5.60 (0.02)	
		2	1	−3	−12.65 (0.02)	
DBT–Mops		1	1	−1	7.80 (0.01)	6.6 × 10^−8^
		1	1	−2	8.20 (0.01)	
		1	1	−3	4.65 (0.01)	
		1	1	−4	−6.20 (0.02)	
		1	1	−2	−13.0 (0.01)

- Log_10_ *β*_011_ of Mes and Mops (tertiary amine), as well as the hydrolysis constants of dibutyltin(IV), decrease linearly with the increase of dioxane proportion in the medium, This may be correlated with the ability of a solvent of relatively low dielectric constant to decrease the electrostatic forces between the proton and ligand anions in the case of ligand dissociation and that between the proton and hydrolyzed form of organotin(IV).
- The variation of the stability constants of the DBT complexes with Mes as a function of solvent composition is shown in Fig. 2. The stability constants of the MLH_−1_ species with Mes and Mops increase with increasing dioxane proportion in the mixed solvent. On the other hand the amount of ML, MLH_−2_, MLH_−3_ species decrease with increasing dioxane proportion in the mixed solvent. This may be explained on the premise that the MLH_−1_ species involves neutralization of charges.

## Conclusion

**Fig. 3:**
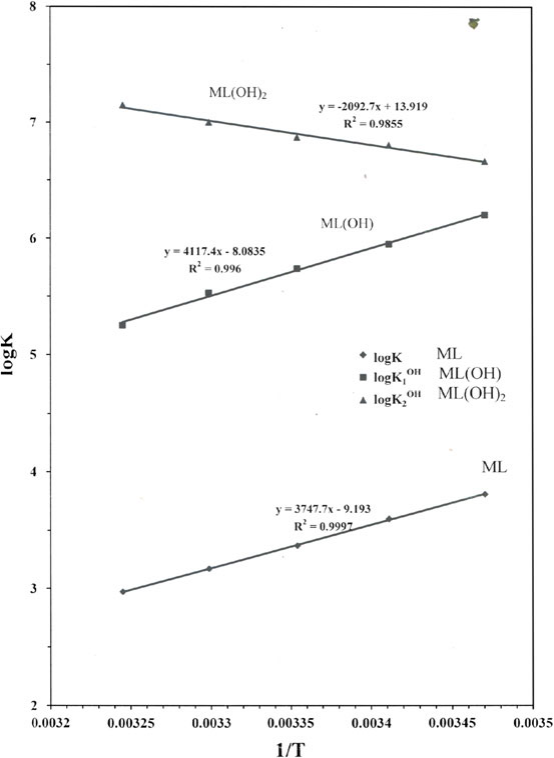
Effect of temperature on log_10_ *K* of the DBT–Mes complex in aqueous solutions

The formation equilibrium of DBT(IV) complexes involving zwitterionic buffer (Mes and Mops) were investigated at different temperatures and variation of water–dioxane proportion. The effect of dioxane as a solvent on the formation constants of DBT–Mes and DBT–Mops complexes decrease linearly with the increase of temperature in the medium (Fig. 3).

## References

[1] Durand S, Sakamoto K, Fukuyama T, Orita A, Otera J, Duthie A, Dakternieks D, Schulte M, Jurkschat K (2000). Cationic organotin clusters for highly efficient alcohol acetylation catalysts. Organometallics.

[2] Omae I (2003). Organotin antifouling paints and their alternatives. Appl. Organomet. Chem..

[3] Miller DP, Craig PJ, Smith PJ (1998). Chemistry of Tin.

[4] Gielen M (2002). Organotin compounds and their therapeutic potential. Appl. Organomet. Chem..

[5] Beckmann J, Jurkschat K (2001). Stannasiloxanes: from rings to polymers. Coord. Chem. Rev..

[6] Chandrasekhar V, Nagendran S, Baskar V (2002). Oragnotin assemblies containing Sn–O bonds. Coord. Chem. Rev..

[7] Good NE, Winget GD, Winter W, Connolly TN, lzawa S, Singh RMM (1966). Hydrogen ion buffers for biological research. Biochemistry.

[8] Good NE, lzawa S (1972). Hydrogen ion buffers. Methods Enzymol..

[9] Ferguson WJ, Braunschweiger KI, Braunschweiger WR, Smith JR, McCormick JJ, Wasmann CC, Jarvis NP, Bell DH, Good NE (1980). Hydrogen ion buffers for biological research. Anal. Biochem..

[10] Gregory JD, Sajdera SW (1970). Economic meaning of a shortage. Science.

[11] Nakon R, Krishnamoorthy CR (1983). Free-metal ion depletion by “Good’s buffers”. Science.

[12] Pope JM, Stevens PR, Angotti MT, Nakon R (1980). Free metal ion depletion by good buffers: II. *N*-(2-acetamido)-2-aminoethanesulfonic acid (ACESH): complexes with calcium(II), magnesium(II), manganese(II), cobalt(II), zinc(II), nickel(II), and copper(II). Anal. Biochem..

[13] Anwar ZM, Azab HA (1999). Ternary complexes in solution. Comparison of the coordination tendency of some biologically important zwitterionic buffers toward the binary complexes of some transition metal ions and some amino acids. J. Chem. Eng. Data.

[14] Azab HA, Deghaidy FS, Orabi AS, Farid NY (2000). Comparison of the effectiveness of various metal ions in the formation of the ternary complexes containing adenosine 5′-mono-, 5′-di-, and 5′-triphosphate and some zwitterionic buffers for biochemical and physiological research. J. Chem. Eng. Data.

[15] Anwar ZA, Azab HA (2001). Role of biologically important zwitterionic buffer secondary ligands in the stability of the ternary complexes containing some metal ions and guanidine 5′-monophosphate, inosine 5′-monophosphate and cytosine 5′-monophosphate. J. Chem. Eng. Data.

[16] Abd-Alla E, Mohamed MMA, Mahmoud MR (2003). Complex formation reactions of dimethyltin(IV) with some zwitterionic buffers. J. Coord. Chem..

[17] Bates RG (1975). Determination of pH: theory and practice.

[18] Shoukry MM, Hosny WM, Khalil MM (1995). Equilibrium and hydrolysis of amino acid esters in mixed–ligand complexes with *N*-iminodiacetate copper (II). Trans. Met. Chem..

[19] Al-Flaijj O, Mohamed MMA, Shehata MM, Shoukry MM (2001). Interaction of dimethyltin(IV) with DNA constituents. Monatsh. für Chem..

[20] Gans P, Sabatini A, Vacca A (1976). An improved computer program for the computation of formation constants from potentiometric data. Inorg. Chem. Acta.

[21] Pettit, L.: Acad. Software (2002)

[22] Surdy P, Rubini P, Buzas N, Henry B, Pellerito L, Gajda T (1999). Interaction of dimethyltin(IV)^2+^ cation with Gly–Gly, Gly–hiss, and some related ligands. A new case of a metal ion able to promote peptide nitrogen deprotonation in aqueous solution. Inorg. Chem..

[23] Natsume T, Aizawa S, Hatano K, Funabshi K (1994). Hydrolysis, polymerization, and structure of dimethyltin(IV) in aqueous solution: molecular structure of the polymer [(SnMe_2_)_2_(OH)_3_]ClO_4_. J. Chem. Soc. Dalton Trans. I.

[24] Buzas N, Gajda T, Nagy L, Kuzmann E, Vertes A, Burger K (1998). Unusual coordination behavior of d-fructose towards dimethyltin; metal-promoted deprotonation of alcoholic OH groups in aqueous solutions of low pH. Inorg. Chim. Acta.

[25] Arena, G., Purrello, R., Rizzarelli, E., Gianguzza, A., Pellerito, L.: Thermodynamics of hydroxo complex formation of dialkyltin(IV) ions in aqueous solution. J. Chem. Soc. Dalton Trans. I 773–777 (1989). doi:10.1039/DT9890000773

[26] Al-Najjar AA, Shehata MR, Mohamed MMA, Shoukry MM (1999). Equilibrium studies of organotin(IV) complexes of peptide. Main Group Met. Chem..

[27] Mash HE, Chin YP, Sigg L, Hari R, Xue H (2003). Complexation of copper by zwitterionic aminosulfonic (Good) buffers. Anal. Chem..

[28] Rees DO (1980). Experimental evolution of the effective dielectric constant of proteins. J. Mol. Biol..

[29] Rogers NK, Roore GR, Strenberg MJE (1985). Electronic interactions in globular proteins: calculation of the pH dependence of the redox potential of cytochrome. J. Mol. Biol..

[30] Sigel H, Martin RM, Tribolet R, Haring UK, Malini-Balakrishran R (1985). An estimation of the equivalent solution dielectric constant in the active-site cavity of metalloenzymes. Eur. J. Biochem..

[31] Åkerlöf G, Short OA (1953). The dielectric constant of dioxane–water mixtures between 0 and 80 degrees: correction. J. Am. Chem. Soc..

[32] Sigel H (1989). Hydrophobic in biological systems: some background information based on ligand–ligand interaction in metal–ion complexes. Pure Appl. Chem..

